# 

**Published:** 1913-03-15

**Authors:** 


### ANNOUNCEMENTS.

Institute of Dental Pedagogics Officers. The officers elected for the ensuing year are: President, Dr. D. H. Squire of Buffalo; Vice-President, Dr. F. W. Gethro, of Chicago; Secretary-Treasuer, Dr. John F. Biddle of Pittsburgh. The next meeting will probably be held at Buffalo.

Dental Societies Meeting in April: Arkansas State at Little Rock, 7, 8 and 9. Connecticut State, at Waterbury 15 and 16. Michigan State, at Grand Rapids, 10, 11 and 12. Faculties Association of American Universities at Boston, 22 and 23.

Texas State Dental Association. The thirty-third annual meeting of the Texas State Dental Association will be held at Temple, Tex., May 15, 16 and 17, 1913. The clinics will in charge of Dr. J. O. Hall Waco, Tex., who will furnish any information relative to same. Exhibitors desiring space will please address Dr. J. M. Murphy, Temple, Tex. All ethical members of the profession are cordially invited to attend the meeting. Any other information will be cheerfully furnished by the secretary.

Guy Morgan, *President*,
Paris, Tex.

J. G. Fife, *Secretary*,
Dallas, Tex.

